# Transformers for Remote Sensing: A Systematic Review and Analysis

**DOI:** 10.3390/s24113495

**Published:** 2024-05-29

**Authors:** Ruikun Wang, Lei Ma, Guangjun He, Brian Alan Johnson, Ziyun Yan, Ming Chang, Ying Liang

**Affiliations:** 1 Beijing Institute of Satellite Information Engineering, Beijing 100095, China; 2 State Key Laboratory of Space-Ground Integrated Information Technology, Space Star Technology Co., Ltd., Beijing 100095, China; 3 Jiangsu Provincial Key Laboratory of Geographic Information Science and Technology, Key Laboratory for Land Satellite Remote Sensing Applications of Ministry of Natural Resources, School of Geography and Ocean Science, Nanjing University, Nanjing 210023, China; 4 Natural Resources and Ecosystem Services, Institute for Global Environmental Strategies, 2108-11, Kamiyamaguchi, Hayama, Kanagawa 240-0115, Japan

**Keywords:** deep learning, convolutional neural network, recurrent neural networks (RNNs), segmentation, classification, change detection, time series, image fusion, object detection

## Abstract

Research on transformers in remote sensing (RS), which started to increase after 2021, is facing the problem of a relative lack of review. To understand the trends of transformers in RS, we undertook a quantitative analysis of the major research on transformers over the past two years by dividing the application of transformers into eight domains: land use/land cover (LULC) classification, segmentation, fusion, change detection, object detection, object recognition, registration, and others. Quantitative results show that transformers achieve a higher accuracy in LULC classification and fusion, with more stable performance in segmentation and object detection. Combining the analysis results on LULC classification and segmentation, we have found that transformers need more parameters than convolutional neural networks (CNNs). Additionally, further research is also needed regarding inference speed to improve transformers’ performance. It was determined that the most common application scenes for transformers in our database are urban, farmland, and water bodies. We also found that transformers are employed in the natural sciences such as agriculture and environmental protection rather than the humanities or economics. Finally, this work summarizes the analysis results of transformers in remote sensing obtained during the research process and provides a perspective on future directions of development.

## 1. Introduction

Since 2014, advances in deep learning (DL) have led to the development of many new remote sensing (RS) image-processing techniques [1]. Convolutional neural networks (CNNs), which have been popular in computer vision (CV), have now become a popular method for RS image-processing tasks like image classification and semantic segmentation. However, when extracting features, the convolutional layer of CNNs is limited to local pixel operations and lacks consideration of global information. This localized constraint often leads to suboptimal solutions in image processing, making it challenging to model global relationships. With the recent popularity of transformers in the field of natural language processing (NLP), this architecture started to be applied to CV. Transformers with global modeling capabilities seem to be the way to address the limitations of CNNs. Major advances were achieved for different tasks, including image recognition [2], object detection [3], and semantic segmentation [4].

The RS field has also witnessed the performance of transformers in RS image processing [5]. However, due to various factors, including the greater number of parameters in remote sensing images compared to natural images [6] and the limited availability of such imagery, research on transformers in RS remains relatively nascent. There are three extant reviews related to the topic, with two focusing more generally on deep learning in RS [7,8] and providing only brief overviews of transformers in RS, and the last, by Aleissaee et al. [9], focusing on RS image types (e.g., hyperspectral imagery, synthetic aperture radar imagery). Based on their review, they discussed the strengths and weaknesses of CNNs and transformers for these different RS image types. However, we believe that the types of processing tasks used in RS are also highly diverse, and thus the relative performance of transformers may vary for these different tasks. Therefore, it is also necessary to analyze the existing research on transformers in RS from the perspective of tasks.

**Figure 1:**
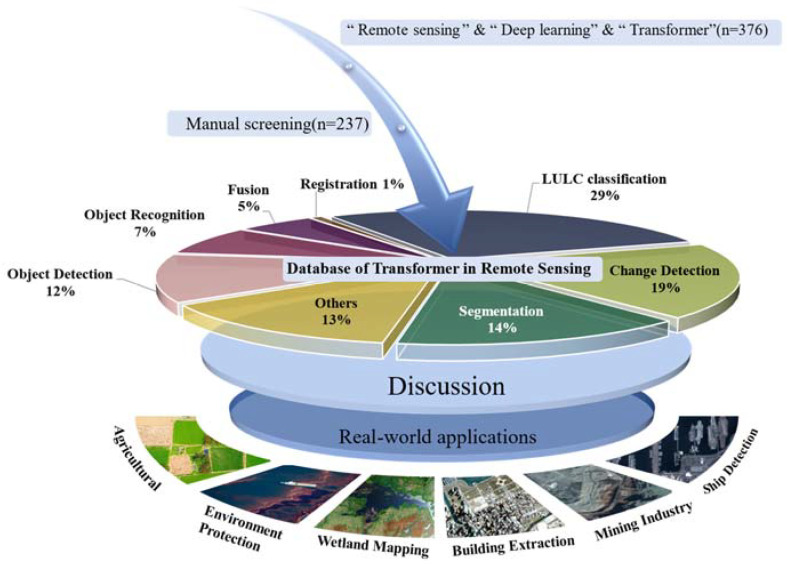
Outline of review, tasks, challenges, and applications.

In this work, through a systematic review of the literature, we explored the strengths and weaknesses of transformers for different RS processing tasks. For this, we developed a database of the literature relating to the usage of transformers for RS image analysis tasks and categorized the data into eight RS image-processing tasks: land use/land cover (LULC) classification, segmentation, fusion, change detection, object detection, object recognition, registration, and “other”. Figure 1 illustrates the outline of this study.

## 2. The Development of Transformers in Image Analysis

### 2.1. The Technology of Transformers

The transformer model was initially proposed by the Google team in 2017 and was first employed for machine translation [10], pioneering the attention mechanism to achieve efficient parallel computation. Before transformers, recurrent neural networks (RNNs) were the preferred architecture for processing sequential data. However, RNNs suffer from the issues of forgetting information over time and relatively slow sequential information processing when dealing with lengthy sequences.

The self-attention mechanism serves as the core of the transformer model. It adeptly captures contextual information for each element within the input sequence, thereby effectively modeling long-range dependencies inherent in the sequence. The standard transformer model comprises four modules: input, output, encoder, and decoder. The encoder and decoder are utilized to map the input and output sequences into high-dimensional spaces, respectively. This is where self-attention mechanisms are computed. The encoder consists of multiple layers with identical structures stacked on top of each other. Each layer comprises a multi-head self-attention mechanism and a feed-forward neural network. Similarly structured as the encoder, the decoder incorporates a self-attention mechanism in each layer based on the output from the encoder. This enables the decoder to focus on global information generated by the encoder for more precise predictions.

Transformers have been found to help overcome the challenges of RNNs and can achieve a higher accuracy than RNNs on extensive datasets, but they tend to overfit on smaller or less diverse datasets [11].

### 2.2. The Development of Transformers in CV

**Figure 2:**
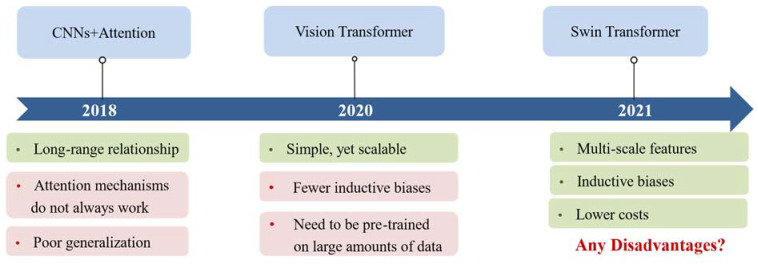
The development process of transformers in CV. The green blocks represent the advantages while the red ones represent the disadvantages.

Transformer-based models have demonstrated a high effectiveness across various NLP tasks and can be easily adapted to new ones [12]. For example, transformers have demonstrated robust modeling capabilities in CV. Since the introduction of the method of combining an attention mechanism with convolutional neural networks (CNNs) by Woo et al. [13], significant advancements have been made in CV. The evolution of transformers in CV has progressed from attention-enhanced CNNs to vision transformer [3] and further to swin transformer [14], as is shown in Figure 2. Furthermore, recent years have witnessed extensive research on large transformer-based models. However, their infrastructure did not change much. Transformers for remote sensing (RS) image analysis, a closely related field to CV, follow a similar developmental trajectory, albeit with a slight lag.

### 2.3. The Development of Transformers in RS

Inspired by advances in CV, RS has also undergone an exploration from CNNs to transformers. CNNs are still widely used as common deep learning architectures in remote sensing image processing. However, the convolution filter size of CNNs limits long-range relationship modeling and further, the performance. To expand the receptive field (which refers to the input region that the neurons of each convolutional layer in a CNN perceive), researchers deepened the network to extract high-level features by multilayer convolution. Each convolution operation throws away some information. Researchers hope that the discarded information is entirely devoid of value. However, it has been found that not all of it lacks usefulness. Therefore, as the network deepens, some useful information will be lost, and the computational complexity will increase. With the advancement of deep learning techniques, dilated convolution [15], spatial pyramid pooling (SPP) [16], the pyramid pooling module (PPM) [17], atrous spatial pyramid pooling (ASPP) [18], and receptive field block (RFB) [19] have been proposed one after another, but the limitations of CNNs have still not been completely solved.

Transformers have demonstrated their ability to alleviate the limitations of CNN architectures in RS, particularly through their global-context modeling capabilities. Consequently, researchers have leveraged the strength of transformers in combination with CNNs for capturing global and local relationships, respectively. In our study, we have summarized five primary approaches that combine the two architectures: (1) the reference framework design [20]; (2) knowledge distillation, with a limited application; (3) series–parallel-splicing-based integration [21]; (4) local substitution-based fusion [22]; (5) multi-level hybridization [23].

While the combination of transformers with CNNs is commonly employed in current RS research, there have also been studies showcasing the advancements of approaches based on pure transformers [24]. Considering the current proliferation of diverse large-scale models based on pure transformers, it is plausible that pure transformer-based neural networks may emerge as a future trend.

## 3. Methods and Data

### 3.1. Data Collection

A systematic literature search was conducted using the Web of Science (WOS) database, which contains papers published in many different international RS journals. We conducted a title/keyword/abstract search using the query ‘“Remote sensing” & “Deep learning” & “Transformer”’, limiting the search results to journal articles and conference papers published between 1 January 2021 and 13 May 2023. A total of 376 publications were retrieved from the query. After an initial screening of the title and abstracts of the retrieved papers (excluding publications unrelated to image processing and some reviews), 237 were identified as relevant to the application of transformers in RS. These remaining publications were included for subsequent analysis.

### 3.2. Data

For the quantitative analysis of transformer-based RS image analysis, a database with 20 fields was constructed based on the 237 articles retrieved from WOS (Appendix A). In addition to general literature identification fields (e.g., journal name, authors, etc.), this database also contained various types of quantitative and qualitative information related to the data and processing tasks used in each paper, including the spatial resolution of the imagery used, training sample size, number of model parameters, and model accuracy (Table 1). From this database, we analyzed the characteristics of the existing research on transformers in RS.

As mentioned in the introduction, for our quantitative analysis, we categorized the collected data into eight RS image-processing tasks: land use/land cover (LULC) classification, segmentation, fusion, change detection, object detection, object recognition, registration, and “other”. In LULC classification, images are classified into different land use types, such as urban, forest, etc. In segmentation, geo-objects in RS images are segmented into individual parts for the better analysis and understanding of their properties and variations. In fusion, multi-source remote sensing data are merged into a unified image in order to better extract the information and features of objects. In change detection, the change in geo-objects is detected to analyze and understand the change trend and influencing factors of ground objects. In object detection, specific objects such as vehicles, buildings, etc., are detected in RS images for tracking and analysis. In object recognition, objects are further classified into predefined categories. In registration, different remote sensing images are registered for downstream tasks.

Then, we calculated the relative performance of transformers as compared with other state-of-the-art methods for each of these tasks, based on the results reported in the literature. It is worth noting that, during the analysis, some of the studies in the database failed to provide information for all fields in Table 1. Hence, in alignment with the specific research objectives, only relevant case studies that expound on the corresponding uncertainties were taken into consideration during our statistical analyses. Therefore, the number of experimental case studies that were used for statistical analyses was, in fact, less than 237.

## 4. Results of Quantitative Analysis

### 4.1. General Statistical Results

**Figure 3:**
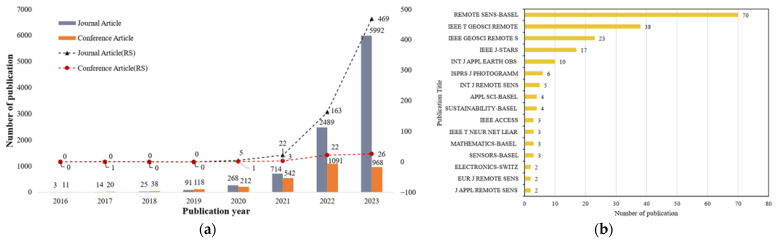
Results of publications. (**a**) Number of conference papers and journal articles in WOS database for general search on [“deep learning” AND “Transformer”]. “RS” denotes “Remote sensing” has been added to the keywords. (**b**) Number of relevant publications per journal.

Before moving to the results of transformers in RS, we first looked at the general trends in the number of papers published on transformers in RS and other research fields (Figure 3a), to understand the rate of progress. From this, we found that the number of papers on transformers is increasing at a rapid rate, both in the field of RS and in general. For example, from 2016–2019, only one conference paper was published on transformers in the field of RS (and no journal articles), but from 2020–2022 (the last year for which we could analyze a full year of papers), the number of conference papers increased from 1 to 22 and the number of journal papers increased from 5 to 192.

Next, considering the 237 papers used for our analysis, we found that 90% (*n* = 214) were journal articles, and the remainder were conference papers (we excluded book chapters and other types of publications from our search). The articles on transformers spanned 35 journals, and 91% (*n* = 195) of the articles were found in 16 journals, detailed in Figure 3b.

**Figure 4:**
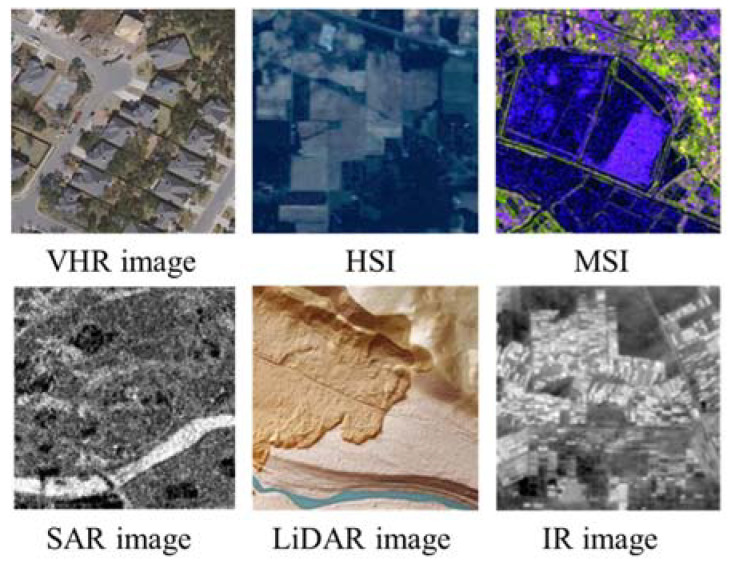
Six types of RS data.

**Figure 5:**
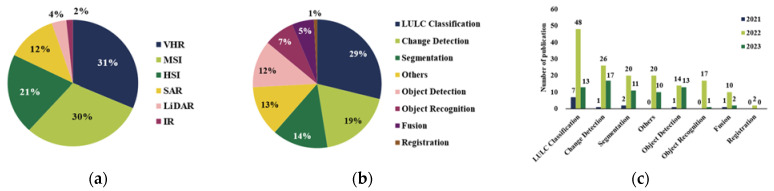
Data and tasks in transformer-based RS image analysis publications database (after manual screening). (**a**) Type of RS image used in publications; (**b**) pie chart of task distribution; (**c**) number of publications each year per task.

By analyzing the utilization of data in publications, our database came to involve six types of RS data: multispectral images (MSIs) (the spectral resolution is in the range of λ/10), hyperspectral images (HSIs) (the spectral resolution is in the range of λ/100), very high-resolution (VHR) images (lack of near-infrared band compared to MSIs), synthetic aperture radar (SAR) images, light detection and ranging (LiDAR), and near-/mid-infrared ray (IR) images, as shown in Figure 4. Among them, the most frequently used is the VHR image (Figure 5a), which is obtained from satellites and aerial imagery datasets, as well as unmanned aerial vehicle (UAV) imagery datasets. In contrast, other types of data are mostly obtained from various satellite platforms, such as Landsat, Sentinel, and GaoFen. Due to the lower cost and easier access, the RS data of Landsat and Sentinel are most frequently used, in addition to the widely used datasets. HSI images are often used for LULC classification, while SAR images and IR images are often used for object detection. There are also digital elevation models (DEMs), meteorological data, crop yield data, etc., which are used in RS.

Figure 5b,c show the relative distribution of the tasks transformers were used for in the RS literature. LULC classification was the most researched task among all publications, while registration is relatively less researched with only two publications in the database. “Other” consisted of various tasks, including spectral reconstruction, RS image captioning, image text retrieval, and RS image denoising, but they were difficult to categorize into these tasks, so we combined them into a single category. Figure 5b,c illustrate the distribution of publications.

**Figure 6:**
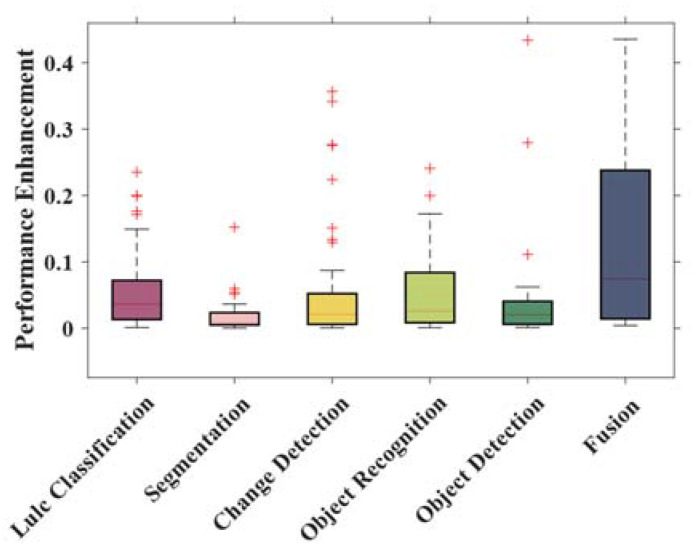
We chose CNNs as a benchmark to calculate the performance enhancement (percentage) of transformer-based methods for different tasks. The red plus signs represent the outlier of the boxplot.

The relative performance of transformer-based methods in each task was next quantitatively investigated. CNN, which widely appeared in comparison methods, was selected as the benchmark for this comparison, to calculate the improvement (percentage) by transformer-based methods, as shown in Figure 6. We found that transformer-based methods have a higher accuracy in fusion and LULC classification tasks, and exhibited a more stable performance (the accuracy distribution is more concentrated) in segmentation and object detection. This stability suggests that the results obtained using transformers for these two tasks can be somewhat anticipated.

### 4.2. Statistical Results in Tasks

**Figure 7:**
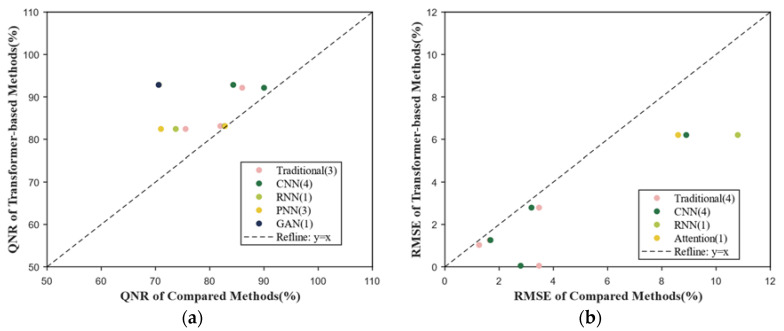
Results in fusion. (**a**) Scatterplot of QNR comparing transformer-based methods and other methods; (**b**) scatterplot of RMSE comparing transformer-based methods and other methods.

**Figure 8:**
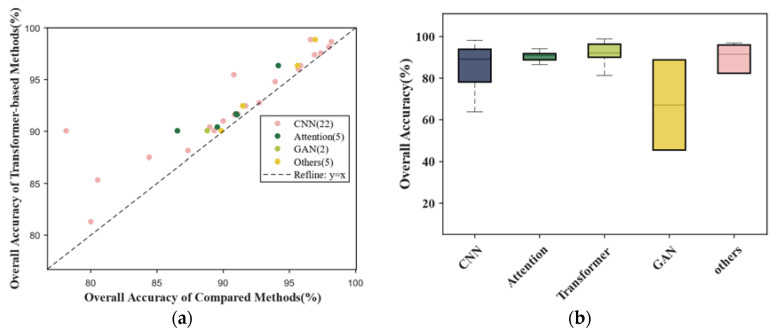
Results in segmentation. (**a**) Scatterplot of OA comparing transformer-based methods and other methods; (**b**) boxplots comparing all methods (line in box represents median).

**Figure 9:**
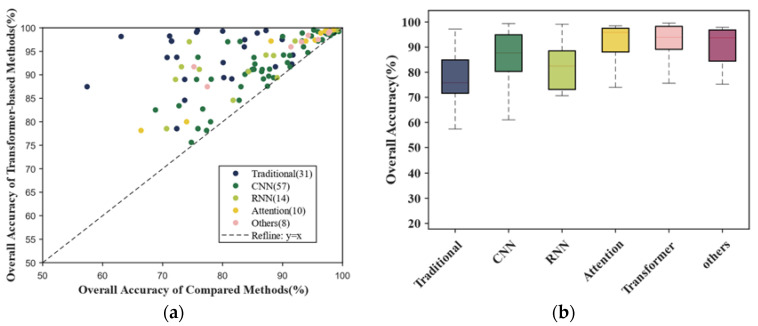
Results in LULC classification. (**a**) Scatterplot comparing transformer-based methods and other methods; (**b**) boxplots comparing all methods (line in box represents median).

**Figure 10:**
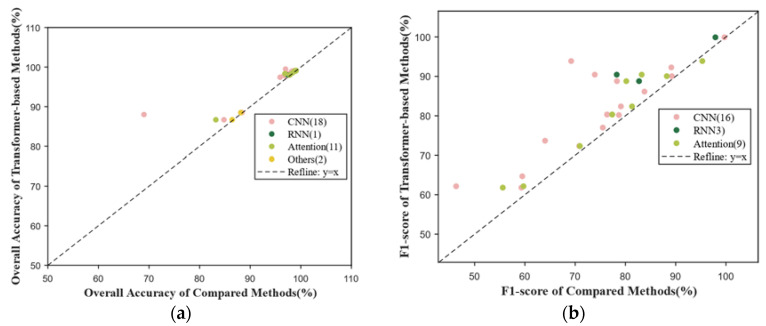
Results in change detection. (**a**) Scatterplot of OA comparing transformer-based methods and other methods; (**b**) scatterplot of F1-score comparing transformer-based methods and other methods.

**Figure 11:**
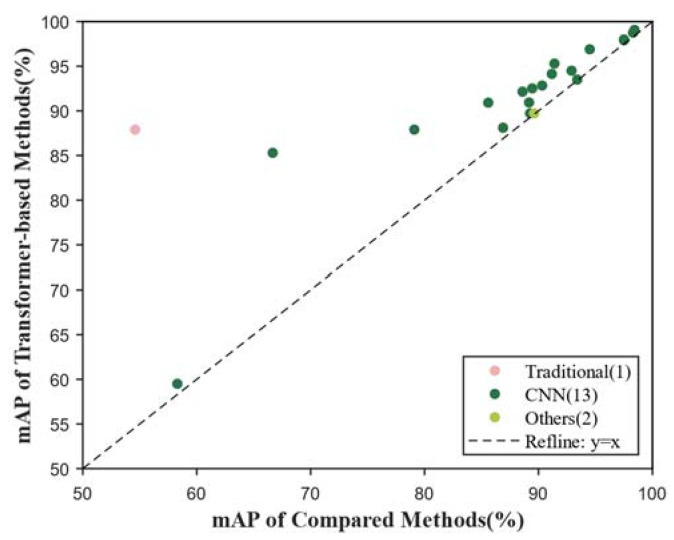
Results in object detection. The scatterplot shows mAP comparing transformer-based methods and other methods.

We chose different evaluation matrices for assessing the accuracy of the transformer and CNN models for different tasks and presented them separately in this subsection. Due to a lack of data, our quantitative analysis did not go into a detailed analysis of every task’s impact. Additionally, the evaluation matrices of deep learning models vary across certain tasks, such as object recognition and registration. Therefore, the statistical results we present do not comprehensively cover all records. In this subsection, we present the results on fusion, segmentation, LULC classification, change detection, and object detection, as depicted in Figure 7, Figure 8, Figure 9, Figure 10 and Figure 11.

#### 4.2.1. Fusion

Figure 7 displays results obtained by analyzing the 14 publications relating to the fusion task. Different from other tasks, the quality with no reference (QNR) and root mean square error (RMSE) are primarily utilized to measure the performance of the models. The QNR is mainly used for panchromatic image sharpening, while the RMSE is utilized for multi-source data fusion. As illustrated in Figure 7, transformers produce higher QNR values for pansharpening compared to alternative methods but result in lower RMSE values for multi-source data fusion. Therefore, it can be inferred that transformers exhibit a significant advantage over alternative architectures in fusion tasks, as evidenced by the scarcity of points along the diagonal line (Figure 7a,b).

#### 4.2.2. Segmentation

Figure 8 displays the results obtained by analyzing the 33 publications relating to segmentation. In segmenting RS images, it is observed that CNN-based methods are more frequently employed than attention-based methods. This observation indicates the irreplaceable role of CNNs in segmentation tasks. In detail, the unique advantages of CNNs in the field of image processing are locality and translation equivariance [25]. Subsequently, the accuracy of various methods was assessed in Figure 8b. Compared to other methods, attention-based and transformer-based methods have higher median values and a reduced variance, which demonstrate superior segmentation accuracy and stability.

#### 4.2.3. LULC Classification

Figure 9 displays the results obtained by analyzing the 47 publications relating to LULC classification. Transformer-based methods had a higher average accuracy and lower mean square error, as shown in Figure 9b. Scatter points representing traditional methods are closer to the upper left in Figure 9a, indicating that the traditional methods perform worse in this task. In contrast, points representing attention are closer to the upper right, which demonstrates the better performance of the attention mechanism. The median OA of attention is also higher than the others, as shown in Figure 9b. Although it is a result of fewer records and higher scores for this method, these figures reflect advancements made by the attention mechanism (including transformer-based methods).

#### 4.2.4. Change Detection

Figure 10 displays the results obtained from analyzing the 44 publications classified as change detection. As demonstrated by empirical studies, CNNs have been extensively employed in change detection tasks [26]. Therefore, we compared the OA in Figure 10a and F1-score in Figure 10b. The scatterplot reveals that attention-based methods exhibit a closer alignment with the diagonal line than CNNs do. This proximity indicates that the performance of attention-based methods is closer to transformer-based methods, since attention mechanisms imitate human attention and generate more discriminative features. So, attention mechanism can enhance the effectiveness of the network when compared with CNNs [27].

#### 4.2.5. Object Detection

Figure 11 displays the results obtained by analyzing the 29 publications relating to object detection. In object detection, CNN-based methods have emerged as dominant, particularly the YOLO family based on CNNs [28]. Therefore, our statistical analysis primarily compares transformers with CNNs. As demonstrated in the scatterplot, a consistently higher accuracy is exhibited by transformer-based methods across all results. However, due to the extensive research maturity associated with CNNs in object detection, several CNN-based methods closely approach the mean average precision (mAP) achieved by transformer-based methods.

Transformer-based methods also outperform other methods in other tasks, and more analysis will be given in the next section.

## 5. A Systematic Review of Transformers in Remote Sensing Image Analysis

### 5.1. Fusion

The fusion task typically consists of two sub-tasks: pansharpening and multi-source information fusion. The objective of pansharpening is to enhance the spatial resolution of multispectral images, while multi-source information fusion aims to integrate remote sensing data from diverse sources to improve the accuracy of downstream tasks. We further categorize multi-source information fusion into distinct forms of data fusion: (1) fusion of RS imagery, observational data, and textual information; (2) fusion of RS imagery from different sensors, such as hyperspectral and SAR images.

Pansharpening refers to obtaining high-resolution multispectral images by fusing panchromatic images and low-resolution multispectral images [29]. In recent years, CNN-based pansharpening methods have exhibited significant advantages over traditional methods. However, both CNN-based and traditional methods tend to treat the two types of data independently and overlook the relationship between multispectral and panchromatic images. Therefore, the performance of the model was limited. Besides connections between two RS images, it is common for objects within a single RS image to exhibit self-similarity. This means that in different locations of the image, there are similar textures and features. This self-similarity allows similar objects to mutually enhance information during the resolution enhancement process. Transformers facilitate information complementation more effectively compared to CNNs [30]. Therefore, Hou et al. devised an ST-based residual self-attention module (STRA), which effectively integrates the advantageous features of the swin transformer and residual neural networks to exploit the inherent self-similarity present in RS images [31].

In multi-source information fusion, the fusion of RS images and observational data for time series analysis has emerged as a prominent research area, particularly for tasks like phenology extraction from multimodal sequence data [32]. Transformers have been proven to be advantageous in processing multimodal and sequential data. Therefore, researchers have started employing transformers to address challenges in the fusion of multimodal RS data. For example, Maillet et al. employed transformers to extract features from satellite image raster data and map-matched weather observations [33]. Then they integrated features over a temporal span to facilitate the prediction of crop diseases. Similarly, Liu et al. utilized a transformer architecture to effectively fuse environmental data (temperature, solar radiation, and precipitation) with time series RS data (the normalized difference vegetation index (NDVI), enhanced vegetation index (EVI), etc.) to achieve accurate long-term crop yield forecasting [34].

Transformers have also been employed for fusing RS images obtained from diverse sensors. Li et al. utilized transformers to process moderate-resolution imaging spectroradiometer (MODIS) data [35], which has a high temporal resolution but limited spatial resolution. They integrated MODIS data with the high-spatial-resolution imagery captured by the LandSat 7 & 8 satellites to achieve a superior-spatiotemporal-resolution composite image. Transformers exhibit advantages in multi-source information fusion due to their inherent mechanism of transforming multi-source data into one-dimensional tokens. This mechanism can bridge the gap between different modalities. However, there is still a challenge when fusing RS images with environmental information: the scarcity of RS images that possess both high temporal and spatial resolutions. To address this issue, we propose leveraging fused high-spatiotemporal-resolution images obtained from different sensors. Subsequently, fused images can be integrated with environmental information to improve the accuracy of downstream tasks. In conclusion, we anticipate more transformer-based studies focusing on multi-source RS information fusion.

### 5.2. Registration

The accuracy of RS image registration significantly impacts the performance of downstream tasks. Despite the commendable performance of CNNs in image registration, certain challenges remain unresolved. Specifically, CNNs struggle to detect features in regions with weak textures within an image. Considering this situation, Yao et al. proposed a perspective-invariant local feature transformer (PILFT) [36]. PILFT combines perspective-invariant correction, CNNs, and attention mechanisms. This novel approach is particularly suitable for images with weak textures and substantial viewpoint changes. Therefore, it has become a valuable complementary method for complex stereo scene matching.

Different from optical images, SAR images pose challenges in accurate annotation due to the presence of speckle noise. Moreover, most DL-based studies on SAR image registration typically focus on small fixed-size patches. These studies may not directly address the matching requirements of wide-strip SAR images. To address this issue, Fan et al. proposed a precise registration method for large-scale SAR images [37]. The method utilizes transformers to handle weak textures during image alignment. The Oriented FAST and Rotated BRIEF operator (ORB) and the Grid-based Motion Statistics method (GMS) are effectively combined to achieve efficient and accurate results.

### 5.3. Segmentation

For dense prediction tasks, researchers have proposed the pyramid vision transformer (PvT) and convolutional vision transformer (CvT) as enhancements to the vision transformer (ViT) model. However, there are still challenges when dealing with complex tasks. In RS image segmentation, there exist several crucial issues: (1) insufficient accuracy in segmenting results and incomplete edge structures; and (2) false alarms and missed pixels caused by environmental noise interference.

In the process of improving edge segmentation accuracy, the fusion of high-resolution features can preserve more complete details of structures and edges. Therefore, it proves advantageous in recovering edge information. For example, Li et al. employed a multi-layer transformer and multi-layer perceptron to integrate features across various scales [38]. Specifically, the multi-layer transformer enhances edge segmentation accuracy through position encoding, while the multi-layer perceptron exhibits increased robustness. This method mitigates the influence of mountainous terrain on built dense areas. Researchers also widely employ edge-guided modeling techniques to enhance edge segmentation outcomes. Xu et al. proposed explicit and implicit edge enhancement transformer models to address the challenges of segmenting object boundaries [39].

To reduce false alarms and missed pixels, researchers have employed transformer-based methods to integrate high-level features with low-level features [40]. Additionally, Wang et al. proposed a coupling structure within CCTNet to correct misclassified regions [41]. This architecture simultaneously utilizes local details extracted by CNNs and global information obtained through transformers, followed by a post-processing method for rectifying misclassified pixels.

### 5.4. LULC Classification

By investigating publications relating to LULC classification, we found that researchers focused their attention on addressing the primary issue of scarce annotated training sample data. Various strategies have been proposed to tackle this problem: (1) utilizing existing large-scale datasets for transfer learning (fine-tuning an unsupervised or self-supervised pre-trained model using labeled data); and (2) augmenting the dataset with generated samples.

For transfer learning, He et al. proposed a method for hyperspectral RS image classification that extracts spectral and spatial features [42]. To address the issue of limited data, they employed VGGNet pre-trained on a large-scale dataset as the initial weight for the spatial feature branch. Meanwhile, Yuan et al. utilized extensive unlabeled datasets for self-supervised pre-training [43]. The findings indicate that deep neural networks with high complexity tend to exhibit overfitting and an unstable performance in the absence of labeled samples. Pre-training significantly enhances the performance of transformers compared to other architectures. Similarly, Yuan et al. adopted a comparable self-supervised pre-training method [44]. And they demonstrated its effectiveness across diverse datasets. Consequently, transfer learning plays a crucial role in advancing transformers.

For data augmentation, various methods have been employed in other studies to generate reliable virtual samples. Newly generated virtual samples have been combined with original samples to form stacked samples [20]. Jamali et al. proposed a 3D generative adversarial network (GAN) to stimulate and increase the number of training data [45]. Certain studies utilize synthetic data as an unsupervised pre-training dataset and subsequently transfer the pre-trained model to address the issue of limited labeled samples [46]. Additionally, Bai et al. successfully achieved few-shot learning for image classification without relying on external datasets [47]. They employed a hybrid architecture of a GAN and transformer encoder, resulting in remarkable accuracy across various datasets.

### 5.5. Change Detection

Current RS change detection approaches face several challenges, including (1) an extreme imbalance between changed and unchanged classes; (2) difficulty in detecting small object changes; and (3) the uneven edges of change regions.

Researchers have proposed various approaches to mitigate the impact of sample imbalance, such as the data balance loss function introduced by Cheng et al. [48]. Additionally, incorporating semantic and pixel-level information into the loss function is another way to address this problem. To tackle the issue of small object change detection, Zhao et al. proposed a novel method that integrates multi-head self-attention for computational efficiency optimization [49]. The method incorporates skip connections to enhance the classifier’s ability to detect small objects. For poor edge segmentation in changed regions, Pang et al. proposed a coordinate attention mechanism [50]. It captures cross-channel information, while incorporating direction-aware and position-sensitive features. This lightweight model enables a more precise localization and identification of target areas. Moreover, Xia et al. proposed a CNN/transformer hybrid model with an edge detection branch [51]. This branch aims to leverage object edges for guiding mask features, thereby improving prediction accuracy for changing region edges. Chen et al. introduced an edge-aware module into EDGE-Net that combines with multi-level transformer architecture to refine features [52].

Furthermore, models based on CNNs and self-attention often overlook the temporal dependencies among features, resulting in “pseudo-change” in complex scenes. To address this issue, transformers were employed to construct tokens representing change intensity and facilitate the interaction of temporal information [53].

In change detection, numerous studies have focused on CNN/transformer hybrid models. For instance, Zhang et al. extensively integrated the distinctive advantages of CNNs and transformers to enhance global representation [54]. A context attention module was constructed using convolutional layers and self-attention mechanisms. In this study, transformers were employed for dual-temporal long-distance context modeling. Additionally, there exist change detection methods solely based on pure transformers, such as CDFormer, proposed by Ding et al. [55], and SwinSUNet, proposed by Zhang et al. [24]. Experimental results from SwinSUNet demonstrated that the inductive bias of CNNs can be partially transferred to transformers. Transformers can focus more attentively on local features.

### 5.6. Object Detection

Compared to natural images, RS images often exhibit densely distributed objects, and there exists a significant semantic relationship among these objects. For example, ships appear in formation, and oil tanks tend to be spatially proximate to ports [56]. Investigating these semantic correlations is currently the focal point of the existing research. The self-attention mechanism in transformers emulates human visual characteristics, so interpretable semantic information can be extracted from feature maps. In contrast, traditional CNN-based object detection methods primarily use the probability of proposal regions and anchor boxes to detect objects. To assess the advantages of transformers in object detection, Detection Transformer (DETR) was proposed as an end-to-end solution [57]. Unlike CNN-based methods, DETR adopts a two-step process involving initial global scanning, followed by the refinement of region proposals to detect targets. It is a logic akin to human behavior when locating objects on a map. DETR has achieved a state-of-the-art mean average precision (mAP) on the COCO dataset. However, no application of DETR in RS has been identified thus far. Hence, we anticipate similar research within this area.

Detecting multi-scale objects in RS images poses a challenging task. Gong et al. combined transformers with YOLO, a CNN-based deep network, aiming to improve object detection results at different scales [58]. They leveraged the global-modeling capabilities of transformers in the neck of the YOLO framework. Among all multi-scale objects, detecting small objects is particularly difficult. To address this issue, researchers have started considering incorporating a multi-scale transformer module following a feature extraction network [59]. The module can enhance the feature extraction ability of the whole model for small objects.

### 5.7. Object Recognition

Object recognition involves classifying objects based on object detection results. Among the research we have investigated, numerous studies have focused on synthetic aperture radar (SAR) image object recognition. Deep learning methods such as CNNs and RNNs often fail to effectively capture the relationship between multi-directional SAR images; so, to address this issue, Li et al. proposed a transformer-based method for SAR image object recognition [60]. The method exploits the relationship between multi-directional SAR image sequences. Additionally, they considered the limited capability of the self-attention mechanism in extracting local features. Therefore, a pre-trained CNN was employed to enhance model accuracy and reduce sample size requirements. Similarly, Xue et al. also developed a hybrid CNN/transformer model [61]. The transformer branch extracts long-term and global features from sequence images, while a 3D CNN serves as a local feature encoder for extracting short-term and local features.

For hyperspectral and multispectral RS image object recognition tasks, image super-resolution techniques can significantly enhance model performance. Gao et al. proposed an aircraft recognition model based on the swin transformer with image super-resolution [62]. This model performed better than other models when evaluated on the MTARSI dataset.

### 5.8. RS Series Data Analysis

#### 5.8.1. Transformers for Spectrum Data Analysis

Transformers were originally proposed for processing sequential data. It is worth noting that spectral data also fall under the category of sequential data. Therefore, researchers leverage the advantages of transformers in extracting spectral features in hyperspectral images. For instance, He et al. designed a two-branch CNN/transformer network for hyperspectral image classification [42]. This network utilized CNN for spatial feature extraction and transformers with skip connections for spectral feature extraction. Yang et al. also investigated the impact of serial fusion and parallel fusion of the spatial branch and spectral branch, respectively [21]. The model they proposed performed well on small samples. Other studies employed two separate transformer branches to process spectral and spatial information. For example, Wang et al. designed a feature aggregation module to fuse the feature information extracted by the two transformer branches [63].

#### 5.8.2. Transformers for Time Series Analysis

In time series analysis, traditional machine learning methods (such as ARIMA, LSTM, etc.) often struggle to capture long-distance relationships. In contrast, transformers perform well at capturing temporal dependence in a time series. This is attributed to the self-attention mechanism and the incorporation of positional encoding. Consequently, transformers have gained popularity for analyzing time series data [64]. However, RS image time series data are typically sparse, since satellites need to wait for the next zenith pass. Therefore, the analysis of RS time series images predominantly takes the form of multi-temporal approaches [65]. The strength of transformers is establishing long-range relationships. In RS time series analysis, this is reflected in the need for long-sequence RS data. Therefore, the primary focus of RS time series studies is on crops. A review of related studies reveals that crops and vegetation are prominent research subjects within RS, due to their distinct seasonal characteristics [34]. And there has been a new research paradigm leveraging contrastive learning methods. This paradigm aims to eliminate pseudo-change caused by phenology factors like season and weather conditions. The intrinsic crop features can be extracted to enable crop classification and yield prediction.

## 6. Challenges

### 6.1. Computational Complexity and Inference Speed of Transformers in RS

**Figure 12:**
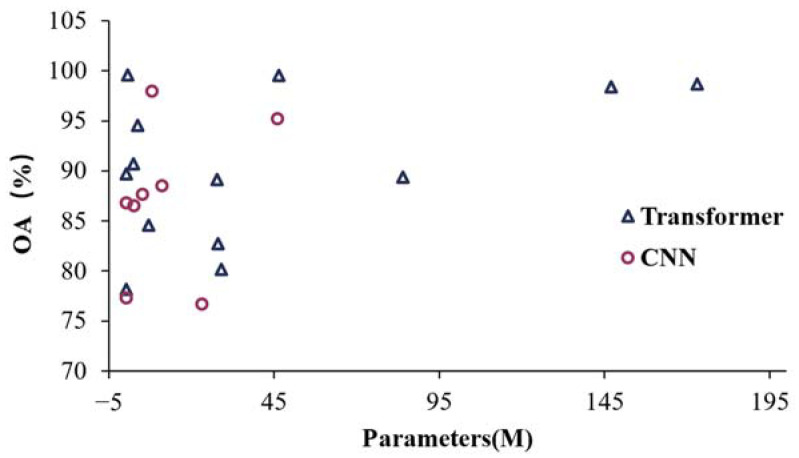
Overall accuracy and parameters of transformers vs. CNNs.

**Figure 13:**
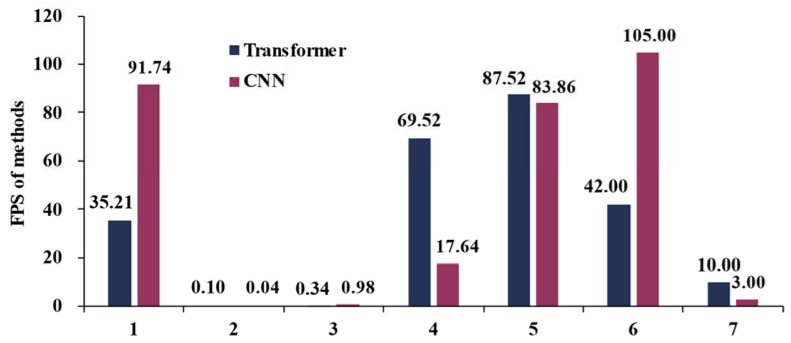
Inference speed of transformers vs. CNNs.

Although transformers offer advantages, their computational complexity poses a significant obstacle to their practical application. Our analysis, supported by statistical data on parameters and inference speed, reveals that transformers require more parameters and inference time compared to CNNs. This finding is illustrated in Figure 12 and Figure 13. The data in Figure 13 are selected according to the following criteria: (1) comparing transformers with CNNs; (2) including inference speed in the evaluation matrix; and (3) FPS is utilized as the evaluation metric. The numerals 1–7 represent each of these seven independent experiments.

Based on the quantitative results presented above, transformers have demonstrated exceptional performance in RS. However, their deep structure and self-attention mechanism often result in a substantial number of model parameters, which limits their applicability in low-configuration environments. To address this limitation, researchers frequently employ various training strategies aimed at reducing the parameters and computational resource consumption. For example, techniques such as model pruning (studies in CV can be found in the work of He et al. [66]) and knowledge distillation (as discussed by Wang et al. [67]), as well as other optimized training strategies, have been adopted. Consequently, certain transformer approaches exhibit fewer parameters than CNNs based on the statistical results presented in this review. Overall, it should be noted that transformers require greater computational resources compared to traditional RS data-processing techniques—particularly during the training phase.

Moreover, deploying the trained transformer model in real-world applications presents its own set of challenges that cannot be overlooked. The inherent complexity and extensive parameters of the model may pose difficulties when deploying it in resource-limited environments. Deploying models in low-configuration environments may encounter issues such as high memory usage and slow inference speed, potentially limiting their usability.

Despite the high inference accuracy of transformers, their inference speed (evaluated by frames per second, FPS) is reduced to varying degrees during the inference stage. To address this issue for real-time application requirements, some researchers have proposed strategies such as employing lightweight MobileViT as the backbone [68]. Statistical findings from this study reveal that, even with various optimization algorithms, transformers can only achieve comparable inference speed to CNNs. In most cases, however, the inference speed of transformers remains significantly lower than that of CNNs, while CNNs exhibit a slightly inferior accuracy (Figure 12). These results indicate that there is still a considerable gap before transformers can be effectively applied in real-time detection for RS images or video analysis.

### 6.2. Large-Scale RS Dataset and Large RS Model

#### 6.2.1. Pre-Training on Large-Scale RS Dataset

**Figure 14:**
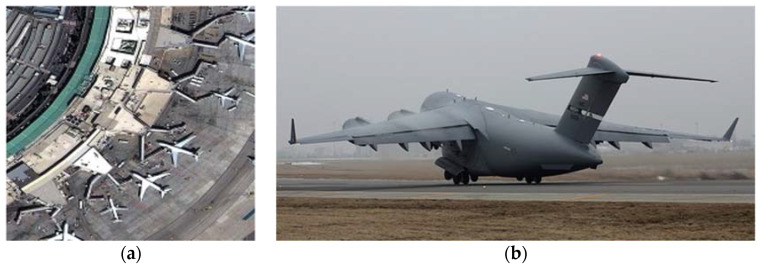
We searched for “plane” in the RS image dataset and the natural image dataset represented by ImageNet. (**a**) Planes in RS image; (**b**) a plane in ImageNet.

It has been observed that ImageNet, which consists of natural images, is the primary dataset used for pre-training models in RS [69]. Although experimental results have shown that pre-training with ImageNet leads to performance improvement, it may not be entirely suitable for processing RS images. The success of transformers on ImageNet can be attributed to their ability to capture global features. However, since objects in RS datasets are typically small-scale, local features become more important. For instance, we compared the representation of “plane” in both the RS image dataset and ImageNet, as depicted in Figure 14. We found that the “plane” only occupies a small area in the former, while occupying almost the entire image in the latter. Therefore, although pre-training with an inappropriate dataset offers advantages over training from scratch, using a more appropriate dataset could potentially lead to higher accuracy.

The accuracy of RS deep learning models can be enhanced by constructing large-scale RS datasets, which have been the focus of recent studies. A novel dataset called SATLASPRETRAIN was proposed [70], demonstrating an average accuracy improvement of 18% compared to the ImageNet fine-tuning across various downstream tasks. Wang et al. trained a series of backbones using MillionAID, the largest existing dataset for RS scene recognition [71]. Experimental results indicated that pre-training with RS data offered a more favorable starting point for fine-tuning than pre-training with ImageNet. Furthermore, they compared the performance of transformer-based models with other architectures and found that transformers exhibit a superior performance. In 2024, some new large-scale RS datasets have been released [72], which will bring new developments to the field of remote sensing.

#### 6.2.2. Transformers and Large Multimodal RS Model

Multimodal RS data can enhance the information content of RS objects, thereby offering significant research potential. However, they also present higher research challenges compared to single-modal data [73]. In CV, transformers have demonstrated advantages in processing multimodal data due to their more general and flexible modeling space [74]. Consequently, researchers started employing transformers to address multimodal problems in RS image text retrieval [75] and RS visual question answering [76]. Currently, there exists a scarcity of large-scale multimodal datasets, leading to researchers’ need to collect multimodal data by themselves. To address this issue, the Globe230k dataset [77] was proposed to improve the quality of training data for RS semantic segmentation. This dataset includes not only RGB bands but also additional features like the normalized vegetation index (NDVI), digital elevation model (DEM), vertical/vertical polarization (VV) band, and vertical/horizontal polarization (VH) band. We anticipate that more multimodal RS datasets will be constructed.

In recent years, the concept of large-scale models has permeated various fields. In natural language processing, ChatGPT has led to significant advancements. In RS, breakthroughs have been achieved with the introduction of pioneering multi-billion-scale vision models for RS data [78], the first generative pre-training model for cross-modal RS data, called RingMo [79], and the development of a groundbreaking multi-billion-scale basic model for RS [80]. These remarkable achievements are all based on the utilization of the vision transformer and swin transformer architectures, reaffirming the superiority of transformers in large-scale multimodal RS data processing.

## 7. Applications

Transformers have been applied in various fields, including agriculture, forestry, meteorology, hydrology, environmental protection, etc. In agriculture, researchers employ transformers for species surveys and crop yield prediction. In the urban context, transformers are primarily used for building extractions in urban regions, as well as for specific area extraction, which serves as an important reference for urban planning and construction. In environmental protection, transformers are used for smoke detection to detect wildfires, building damage assessment, and melt pond detection. Transformers play an important role in disaster area assistance and ecological environment protection. In mapping, there are several applications such as wetland mapping and urban area mapping. Transformers not only improve the accuracy of mapping but also enhance the utilization of RS data through the fusion of different types of data, including satellite data and weather data [81]. In geology, transformers provide a solution for monitoring tailings pond detection [82]. In other applications, transformers are primarily employed for ship/aircraft detection and recognition and RS image captioning, as well as exploring deep learning techniques. Transformers possess significant potential and value for both military and commercial use.

**Figure 15:**
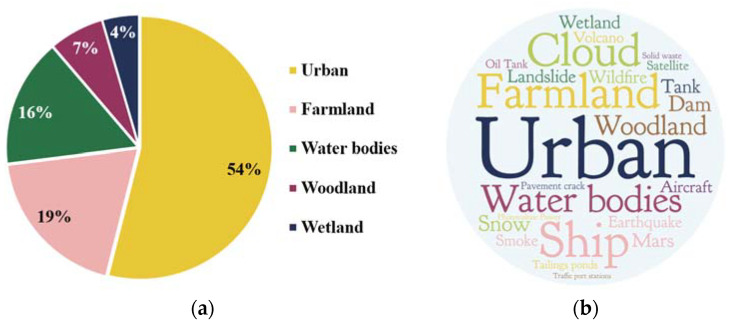
Results of applications of transformers in RS. (**a**) Pie chart involving articles that use data from single scene; (**b**) study target cloud of articles in (**a**).

The most widely used scene for transformers in RS is currently urban areas, as evidenced by the count of publications in the database. Figure 15a illustrates the weight of each study target in the database, while Figure 15b visually represents the highest-frequency terms appearing in the title and abstract of the peer-reviewed literature, with a larger font size indicating higher frequencies. Table 2 showcases typical RS applications of transformers in each field.

Due to the continuous exploration of transformers by researchers, they have been applied quite successfully in different scenarios of RS, playing different roles. However, we found that the current research of transformers in RS is only limited to observation related to the environment, whether it is the natural environment or urban environment. There is a lack of research in the fields of humanities, economy, and finance. For instance, urban informal areas have a close connection to the urban development, and in-depth research is supported by earth observation data. We anticipate that the utilization of transformers will broaden, as RS image acquisition techniques advance further and RS data continue to be abundant.

## 8. Conclusions

Using information extracted from 237 scientific publications, in this study we conducted a systematic review of the use of transformers in RS, and a quantitative analysis of transformers’ relative performance (compared with other state-of-the-art methods like CNNs), considering seven common RS image-processing tasks. Along with the quantitative results, we discussed the status and analyzed the challenges of transformer-based deep learning methods in RS image analysis. Subsequently, the applications of transformers in RS were presented. Although the limited number of records prevented a detailed statistical analysis on the effect of each contributing factor, generally we can see the following:

1. Transformers have a generally better performance than other architectures in different tasks in RS image analysis, with the results of the quantitative analysis validating the potential of the attention mechanism and transformers.
2. Transformers have advantages in LULC classification and fusion, with a more stable performance in segmentation and object detection.
3. In most research, transformers have been combined with other architectures such as CNNs to improve the model accuracy, while some pure transformer models also showed their potential.
4. Transformers perform well in multimodal data processing and complex feature capture, with the ability to aggregate features across different feature spaces, as well as globally, which is not the only solution to this type of problem but is still very important and has great potential for development.
5. In time series analysis, researchers have developed a research paradigm that differs from machine learning methods by eliminating the effect of weather on crops and focusing solely on crop characteristics. This enables higher-accuracy tasks such as crop classification and crop yield prediction. Transformers play an essential role in this analysis of long time series.
6. Researchers have encountered the challenges of the high computational complexity and computation times of transformers. New techniques such as improving the range of the attention value and calculation formulas have been proposed to simplify the computational process of transformers in RS.
7. Transformers have been explored in a wide range of scenes, including urban, farmland, woodland, etc. But we found that transformers are solely employed on earth observation. There is a lack of research in the fields of humanities, economy, and finance.

As previously noted, by adopting some of the best architectural designs (pyramid structure, inductive bias, residual connectivity, etc.) and training strategies (knowledge distillation) from CNNs, researchers aim to integrate the strengths of CNNs and transformers into a model. Transformers also perform well in multi-temporal image analysis tasks, with their multimodal fusion capability and great sequence-feature capture capability. Throughout all the publications we researched, there are some problems behind transformers’ excellent performance that need to be solved. Large-scale standard remote sensing image datasets are needed in RS. Although there have been recent releases of large-scale remote sensing datasets [70], most of the existing studies using pre-training are based on ImageNet, which does not exactly match remote sensing tasks. In network architecture, despite a series of improvements being proposed, massive calculations and a slow inference speed are still problems faced by researchers. Possible solutions are to improve the calculation of the attention value and to reduce the range of it. How to reduce the complexity of the calculation, and at the same time take into account high accuracy, is a future research direction. Finally, the ultimate goal of RS research is to provide decision support for human behavior, not only at the technique level. We recommend that research on transformers in the humanities, economics, finance, and other fields be conducted to employ their strengths for the advancement of these fields.

## Figures and Tables

**Table 1:** Checklist of items used when constructing analysis database for transformers in remote sensing.

Index	Fields	Definition	Type	Categories
1	Title	Title of publication	Free text	
2	Authors	Author	Free text	
3	Year	Publication year	Free text	2021; 2022; 2023
4	Publication type	Type of publications	Classes	Journal Article; Conference Paper
5	Citations	No. of citations by other publications	Numeric	
6	Image resolution	The ground range for a pixel	Numeric	
7	Training sample	Proportion of training data to all data	Numeric	
8	Patch size	Size of the image when entering the network	Numeric	
9	Site type	Type of study area or target	Classes	Urban; Wetland; Farmland; Woodland; Water bodies; Others
10	Task	Tasks in remote sensing	Classes	LULC Classification; Segmentation; Fusion; Change Detection; Object Detection; Object Recognition; Registration; Others
11	Evaluation criteria	Accuracy assessment index	Classes	OA; F1-score; RMSE; IoU; QNR; mAP
12	Accuracy value	Best accuracy value	Numeric	
13	Class number	Number of classes	Numeric	
14	Processing unit	Basis of the classifier	Classes	Pixel; Object; Scene
15	Pre-processing	Methods of pre-processing data	Free text	
16	Parameters	Number of parameters in model	Numeric	
17	FLOPs	Number of calculations required by model	Numeric	
18	Inference speed	Number of images that can be processed per second	Numeric	

**Table 2:** Typical applications of transformers in RS.

Field of Application	Application
Agriculture	Crop type mapping [83]
	Rice yield prediction [34]
	Downy mildew disease detection [33]
	Mariculture cage segmentation [84]
Environment protection	Smoke-like scenes classification [85]
	Building damage assessment [86]
	Detection of melt ponds on sea ice [87]
	Oil spills detection [88]
	Deforestation monitoring [89]
	Snowmelt flood susceptibility assessment [90]
	Tailings ponds detection [82]
Mapping	Wetland mapping [45]
Urban planning	Urban building classification [43,91]
	UIS classification [23,92]
Others	Small object detection [58]
	Ship detection [93]
	RS image captioning [94]

## Appendix A

The database of the manuscript has been uploaded to Gitee, visible at https://gitee.com/jiaozhuwang/transformers_for_-remote_-sensing_-a_-systematic_-review_and_-analysis/blob/master/database.xlsx (accessed on 14 May 2024).
